# (α*R*,4*R*,4a*R*,6a*S*,7*R*,8*S*,10*R*,11*S*)-Methyl α-acet­oxy-4-(3-furan­yl)-10-hy­droxy-4a,7,9,9-tetra­methyl-2,13-dioxo-1,4,4a,5,6,6a,7,8,9,10,11,12-dodeca­hydro-7,11-methano-2*H*-cyclo­octa­[*f*][2]benzopyran-8-acetate (6-*O*-acetyl­swietenolide) from the seeds of *Swietenia macrophylla*

**DOI:** 10.1107/S1600536810039942

**Published:** 2010-10-13

**Authors:** Bey Hing Goh, Habsah Abdul Kadir, Sri Nurestri Abdul Malek, Seik Weng Ng

**Affiliations:** a Institute of Biological Sciences, University of Malaya, 50603 Kuala Lumpur, Malaysia; b Department of Chemistry, University of Malaya, 50603 Kuala Lumpur, Malaysia

## Abstract

The mol­ecule of *O*-acetyl­swietenolide, C_29_H_36_O_9_, isolated from the seeds of *Swietenia macrophylla*, features four six-membered rings connected together in the shape of a bowl; one of the inner rings adopts a twisted chair conformation owing to the C=C double bond. The furyl substitutent is connected to an outer ring, and it points away from the bowl cavity. The hy­droxy group is connected to a carbonyl O atom of an adjacent mol­ecule by an O—H⋯O hydrogen bond, generating a chain running along the *b* axis.

## Related literature

For the absolute stereochemistry assignment, see: Bickii *et al.* (2000 ▶); Kadota *et al.* (1990 ▶); Mootoo *et al.* (1999 ▶); Narender *et al.* (2008 ▶). For another swietenolide isolated from *Swietenia macrophylla*, see: Goh *et al.* (2010 ▶).
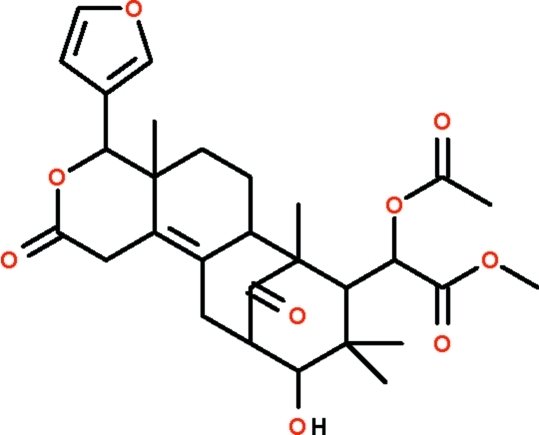


## Experimental

### 

#### Crystal data

C_29_H_36_O_9_
*M* *_r_* = 528.58
Monoclinic, 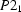

*a* = 11.5648 (9) Å
*b* = 8.4355 (6) Å
*c* = 14.5082 (11) Å
β = 112.985 (1)°
*V* = 1302.98 (17) Å^3^
*Z* = 2
Mo *K*α radiation
μ = 0.10 mm^−1^
*T* = 100 K
0.35 × 0.15 × 0.05 mm

#### Data collection

Bruker SMART APEX diffractometer
12419 measured reflections
3178 independent reflections
2952 reflections with *I* > 2σ(*I*)
*R*_int_ = 0.034

#### Refinement

*R*[*F* ^2^ > 2σ(*F* ^2^)] = 0.034
*wR*(*F* ^2^) = 0.100
*S* = 1.00
3178 reflections
353 parameters
2 restraints
H atoms treated by a mixture of independent and constrained refinement
Δρ_max_ = 0.27 e Å^−3^
Δρ_min_ = −0.20 e Å^−3^

### 

Data collection: *APEX2* (Bruker, 2009 ▶); cell refinement: *SAINT* (Bruker, 2009 ▶); data reduction: *SAINT*; program(s) used to solve structure: *SHELXS97* (Sheldrick, 2008 ▶); program(s) used to refine structure: *SHELXL97* (Sheldrick, 2008 ▶); molecular graphics: *X-SEED* (Barbour, 2001 ▶); software used to prepare material for publication: *publCIF* (Westrip, 2010 ▶).

## Supplementary Material

Crystal structure: contains datablocks global, I. DOI: 10.1107/S1600536810039942/bt5372sup1.cif

Structure factors: contains datablocks I. DOI: 10.1107/S1600536810039942/bt5372Isup2.hkl

Additional supplementary materials:  crystallographic information; 3D view; checkCIF report

## Figures and Tables

**Table 1:** Hydrogen-bond geometry (Å, °)

*D*—H⋯*A*	*D*—H	H⋯*A*	*D*⋯*A*	*D*—H⋯*A*
O5—H5⋯O8^i^	0.84 (1)	1.99 (1)	2.827 (2)	175 (3)

Symmetry code: (i) 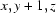.

**Fig. 1.**
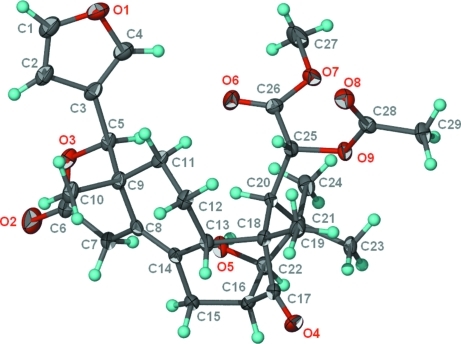
Anisotropic displacement ellipsoid plot (Barbour, 2001) of C29H36O9 at the 70% probability level; hydrogen atoms are drawn as spheres of arbitrary radius.

## supplementary crystallographic information

### Comment

*Sweietenia macrophylla* is a large mahogany tree growing in the rainforests of Malaysia. The extracts of the seeds contain flavonoids, saponins and alkaloids that are commecialized in local herbal products. A previous study reports the crystal structure of swietenolide diactate (Goh *et al.*, 2010). The title compound (Scheme I, Fig. 1), which was isolated from the same plant, differs only in having a hydroxy group in place of a acetoxy group. The hydroxy group engages in O–H···O hydrogen bonding to generate a chain structure.

### Experimental

The finely ground seeds of*Sweietenia macrophylla* (600 g) were soaked in ethanol at room temperature for three days. The mixture was filtered and the solvent evaporated to give a dark yellow crude material (70 g). A portion (40 g) was successively extracted with *n*-hexane, ethyl acetate and water to give an *n*-hexane-insoluble residue. The residue was partitioned between ethyl acetate-water (1:1) to give an ethyl acetate-soluble fraction (30 g, 80%).

This fraction (3 g) was subjected to column chromatography on silica gel (70–230 mesh, 300 g), with initial elution by *n*-hexane, followed by increasing proportions of chloroform. Eleven fractions were obtained. The fourth fraction (2 g) was further subjected to column chromatography (70–230 mesh,200 g), initially eluting with *n*-hexane and later with acetone to give twelve fractions.

The eighth fraction (600 mg) was dissolved in methanol and kept in a refrigerator. A white solid was obtained after two days, and a second crop was obtained after another two days. Recrystallization of the first crop from chloroform yielded colorless crystals of the swietenolide diacetate (yield 15 mg). The ninth fraction (80 mg) yielded *O*-acetylswietenolide (13 mg) after recrystalization from chloroform.

### Refinement

Carbon-bound H atoms were placed in calculated positions (C—H 0.95 to 1.00 Å) and were included in the refinement in the riding model approximation, with *U*(H) set to 1.2 to 1.5*U*(C). The hydroxy H-atom was located in a difference Fourier map, and was refined isotropically with a distance restraint of O–H 0.84±0.01 Å.

2201 Friedel pairs were merged.

### Crystal data

**Table:**

C_29_H_36_O_9_	*F*(000) = 564
*M**_r_* = 528.58	*D*_x_ = 1.347 Mg m^−^^3^
Monoclinic, *P*2_1_	Mo *K*α radiation, λ = 0.71073 Å
Hall symbol: P 2yb	Cell parameters from 4876 reflections
*a* = 11.5648 (9) Å	θ = 2.9–28.1°
*b* = 8.4355 (6) Å	µ = 0.10 mm^−^^1^
*c* = 14.5082 (11) Å	*T* = 100 K
β = 112.985 (1)°	Prism, colorless
*V* = 1302.98 (17) Å^3^	0.35 × 0.15 × 0.05 mm
*Z* = 2	

### Data collection

**Table:**

Bruker SMART APEX diffractometer	2952 reflections with *I* > 2σ(*I*)
Radiation source: fine-focus sealed tube	*R*_int_ = 0.034
graphite	θ_max_ = 27.5°, θ_min_ = 1.9°
ω scans	*h* = −15→15
12419 measured reflections	*k* = −10→10
3178 independent reflections	*l* = −18→18

### Refinement

**Table:**

Refinement on *F*^2^	Primary atom site location: structure-invariant direct methods
Least-squares matrix: full	Secondary atom site location: difference Fourier map
*R*[*F*^2^ > 2σ(*F*^2^)] = 0.034	Hydrogen site location: inferred from neighbouring sites
*wR*(*F*^2^) = 0.100	H atoms treated by a mixture of independent and constrained refinement
*S* = 1.00	*w* = 1/[σ^2^(*F*_o_^2^) + (0.0733*P*)^2^ + 0.1429*P*] where *P* = (*F*_o_^2^ + 2*F*_c_^2^)/3
3178 reflections	(Δ/σ)_max_ = 0.001
353 parameters	Δρ_max_ = 0.27 e Å^−^^3^
2 restraints	Δρ_min_ = −0.20 e Å^−^^3^

### Fractional atomic coordinates and isotropic or equivalent isotropic displacement parameters (Å^2^)

**Table:**

	*x*	*y*	*z*	*U*_iso_*/*U*_eq_	
O1	0.61851 (17)	0.5009 (2)	1.01707 (12)	0.0283 (4)	
O2	0.47929 (19)	1.1423 (2)	0.73073 (15)	0.0344 (4)	
O3	0.55291 (15)	0.92107 (19)	0.81247 (12)	0.0198 (3)	
O4	0.87219 (16)	0.6373 (2)	0.44745 (12)	0.0257 (4)	
O5	0.89022 (14)	0.99908 (19)	0.69927 (12)	0.0200 (3)	
H5	0.945 (2)	1.059 (3)	0.7393 (18)	0.024 (7)*	
O6	0.92901 (15)	0.6390 (2)	0.91487 (12)	0.0264 (4)	
O7	1.12489 (15)	0.5429 (2)	0.96568 (11)	0.0224 (4)	
O8	1.06597 (15)	0.21550 (19)	0.82808 (12)	0.0218 (3)	
O9	1.10687 (14)	0.46053 (18)	0.78928 (12)	0.0169 (3)	
C1	0.4977 (2)	0.5532 (3)	0.98089 (18)	0.0268 (5)	
H1	0.4365	0.5202	1.0054	0.032*	
C2	0.4760 (2)	0.6575 (3)	0.90604 (17)	0.0237 (5)	
H2	0.3990	0.7097	0.8688	0.028*	
C3	0.5928 (2)	0.6741 (3)	0.89368 (16)	0.0184 (4)	
C4	0.6748 (2)	0.5781 (3)	0.96307 (17)	0.0252 (5)	
H4	0.7605	0.5660	0.9730	0.030*	
C5	0.61902 (19)	0.7714 (3)	0.81867 (16)	0.0162 (4)	
H5A	0.7112	0.7937	0.8451	0.019*	
C6	0.5376 (2)	1.0223 (3)	0.73623 (17)	0.0200 (4)	
C7	0.5993 (2)	0.9818 (3)	0.66575 (16)	0.0174 (4)	
H7A	0.6814	1.0370	0.6892	0.021*	
H7B	0.5471	1.0261	0.5993	0.021*	
C8	0.62213 (18)	0.8084 (3)	0.65186 (15)	0.0144 (4)	
C9	0.57977 (19)	0.6948 (3)	0.71383 (16)	0.0156 (4)	
C10	0.4363 (2)	0.6743 (3)	0.66379 (17)	0.0203 (5)	
H10A	0.4133	0.6334	0.5957	0.030*	
H10B	0.3955	0.7770	0.6610	0.030*	
H10C	0.4088	0.5995	0.7028	0.030*	
C11	0.6478 (2)	0.5347 (2)	0.72606 (16)	0.0159 (4)	
H11A	0.7348	0.5460	0.7762	0.019*	
H11B	0.6046	0.4553	0.7517	0.019*	
C12	0.6513 (2)	0.4750 (3)	0.62825 (16)	0.0178 (4)	
H12A	0.5646	0.4503	0.5813	0.021*	
H12B	0.7002	0.3751	0.6415	0.021*	
C13	0.70907 (19)	0.5924 (3)	0.57788 (16)	0.0159 (4)	
H13	0.6677	0.5706	0.5044	0.019*	
C14	0.67811 (19)	0.7634 (3)	0.59133 (15)	0.0148 (4)	
C15	0.7178 (2)	0.8800 (3)	0.53024 (16)	0.0183 (4)	
H15A	0.7045	0.9891	0.5493	0.022*	
H15B	0.6638	0.8655	0.4585	0.022*	
C16	0.8571 (2)	0.8611 (3)	0.54462 (17)	0.0186 (4)	
H16	0.8712	0.9238	0.4914	0.022*	
C17	0.8692 (2)	0.6869 (3)	0.52527 (16)	0.0189 (4)	
C18	0.85658 (19)	0.5781 (3)	0.60545 (16)	0.0153 (4)	
C19	0.8895 (2)	0.4080 (3)	0.58576 (17)	0.0189 (4)	
H19A	0.8320	0.3742	0.5189	0.028*	
H19B	0.8813	0.3364	0.6361	0.028*	
H19C	0.9760	0.4050	0.5897	0.028*	
C20	0.93657 (19)	0.6467 (2)	0.71258 (15)	0.0125 (4)	
H20	0.8750	0.7049	0.7329	0.015*	
C21	1.03242 (19)	0.7743 (3)	0.71073 (16)	0.0154 (4)	
C22	0.9545 (2)	0.9152 (3)	0.64803 (17)	0.0185 (4)	
H22	1.0139	0.9906	0.6361	0.022*	
C23	1.1253 (2)	0.7223 (3)	0.66323 (18)	0.0211 (5)	
H23A	1.1843	0.6442	0.7067	0.032*	
H23B	1.1719	0.8149	0.6555	0.032*	
H23C	1.0785	0.6748	0.5975	0.032*	
C24	1.1129 (2)	0.8349 (3)	0.81604 (17)	0.0198 (5)	
H24A	1.1701	0.7508	0.8541	0.030*	
H24B	1.0582	0.8656	0.8503	0.030*	
H24C	1.1618	0.9270	0.8110	0.030*	
C25	0.99178 (19)	0.5175 (2)	0.79253 (15)	0.0146 (4)	
H25	0.9310	0.4271	0.7753	0.018*	
C26	1.0111 (2)	0.5757 (3)	0.89756 (16)	0.0179 (4)	
C27	1.1475 (3)	0.5980 (4)	1.06570 (19)	0.0352 (7)	
H27A	1.2382	0.6054	1.1047	0.053*	
H27B	1.1105	0.5233	1.0982	0.053*	
H27C	1.1092	0.7027	1.0620	0.053*	
C28	1.1339 (2)	0.3050 (3)	0.80775 (16)	0.0166 (4)	
C29	1.2531 (2)	0.2641 (3)	0.79648 (18)	0.0216 (5)	
H29A	1.2356	0.2438	0.7258	0.032*	
H29B	1.2898	0.1691	0.8359	0.032*	
H29C	1.3123	0.3526	0.8204	0.032*	

### Atomic displacement parameters (Å^2^)

**Table:**

	*U*^11^	*U*^22^	*U*^33^	*U*^12^	*U*^13^	*U*^23^
O1	0.0289 (9)	0.0383 (10)	0.0209 (8)	−0.0019 (8)	0.0133 (7)	0.0061 (7)
O2	0.0402 (11)	0.0271 (9)	0.0458 (11)	0.0131 (9)	0.0274 (9)	0.0032 (9)
O3	0.0224 (8)	0.0193 (7)	0.0225 (8)	−0.0008 (6)	0.0140 (7)	−0.0051 (6)
O4	0.0284 (9)	0.0365 (9)	0.0172 (8)	0.0106 (8)	0.0142 (7)	0.0037 (7)
O5	0.0157 (7)	0.0171 (7)	0.0276 (9)	0.0008 (6)	0.0090 (7)	0.0000 (6)
O6	0.0182 (8)	0.0465 (10)	0.0158 (8)	0.0061 (8)	0.0079 (6)	−0.0018 (8)
O7	0.0201 (8)	0.0307 (9)	0.0139 (7)	0.0063 (7)	0.0038 (6)	0.0016 (7)
O8	0.0229 (8)	0.0159 (7)	0.0267 (9)	0.0003 (6)	0.0097 (7)	0.0020 (7)
O9	0.0141 (7)	0.0146 (7)	0.0230 (8)	0.0029 (6)	0.0084 (6)	0.0025 (6)
C1	0.0251 (11)	0.0347 (13)	0.0254 (12)	−0.0046 (10)	0.0150 (10)	−0.0012 (10)
C2	0.0208 (11)	0.0304 (12)	0.0249 (12)	−0.0033 (10)	0.0142 (9)	−0.0012 (10)
C3	0.0196 (10)	0.0231 (11)	0.0150 (10)	−0.0037 (9)	0.0096 (8)	−0.0058 (8)
C4	0.0219 (11)	0.0363 (13)	0.0198 (11)	−0.0019 (10)	0.0105 (9)	0.0025 (10)
C5	0.0134 (9)	0.0197 (10)	0.0170 (10)	−0.0018 (8)	0.0077 (8)	−0.0030 (8)
C6	0.0172 (10)	0.0220 (10)	0.0223 (11)	0.0000 (9)	0.0095 (9)	−0.0044 (9)
C7	0.0158 (10)	0.0195 (10)	0.0182 (10)	0.0031 (8)	0.0081 (8)	0.0009 (8)
C8	0.0081 (9)	0.0189 (10)	0.0132 (10)	0.0018 (7)	0.0010 (7)	−0.0003 (8)
C9	0.0116 (9)	0.0203 (10)	0.0151 (9)	−0.0016 (8)	0.0053 (8)	−0.0032 (8)
C10	0.0137 (10)	0.0282 (12)	0.0199 (10)	−0.0035 (9)	0.0076 (8)	−0.0071 (9)
C11	0.0161 (9)	0.0175 (10)	0.0167 (10)	−0.0035 (8)	0.0094 (8)	−0.0018 (8)
C12	0.0165 (10)	0.0189 (10)	0.0200 (10)	−0.0024 (8)	0.0091 (8)	−0.0044 (8)
C13	0.0116 (9)	0.0201 (10)	0.0152 (10)	0.0017 (8)	0.0045 (8)	−0.0032 (8)
C14	0.0105 (9)	0.0203 (10)	0.0124 (9)	0.0026 (8)	0.0031 (7)	0.0008 (8)
C15	0.0147 (10)	0.0249 (10)	0.0156 (10)	0.0057 (9)	0.0063 (8)	0.0051 (9)
C16	0.0183 (11)	0.0241 (11)	0.0182 (10)	0.0055 (8)	0.0123 (9)	0.0081 (9)
C17	0.0136 (10)	0.0274 (11)	0.0172 (10)	0.0050 (8)	0.0077 (8)	0.0045 (9)
C18	0.0131 (9)	0.0193 (10)	0.0146 (9)	0.0019 (8)	0.0066 (8)	0.0007 (8)
C19	0.0188 (10)	0.0228 (11)	0.0174 (10)	0.0027 (9)	0.0096 (9)	−0.0033 (8)
C20	0.0108 (8)	0.0141 (9)	0.0127 (9)	0.0029 (7)	0.0045 (7)	0.0010 (7)
C21	0.0129 (9)	0.0161 (9)	0.0201 (10)	0.0013 (8)	0.0096 (8)	0.0029 (8)
C22	0.0154 (10)	0.0159 (10)	0.0260 (11)	0.0030 (8)	0.0102 (9)	0.0068 (8)
C23	0.0134 (10)	0.0234 (10)	0.0303 (12)	0.0039 (8)	0.0126 (9)	0.0074 (9)
C24	0.0148 (10)	0.0168 (10)	0.0259 (12)	−0.0007 (8)	0.0058 (9)	0.0002 (8)
C25	0.0127 (9)	0.0145 (9)	0.0173 (10)	0.0012 (8)	0.0065 (8)	0.0014 (8)
C26	0.0169 (10)	0.0218 (10)	0.0157 (10)	−0.0007 (9)	0.0071 (8)	0.0028 (8)
C27	0.0271 (13)	0.0530 (18)	0.0161 (11)	0.0144 (13)	−0.0017 (10)	−0.0026 (11)
C28	0.0178 (10)	0.0147 (10)	0.0139 (10)	0.0019 (8)	0.0026 (8)	−0.0014 (8)
C29	0.0183 (10)	0.0187 (10)	0.0276 (12)	0.0055 (9)	0.0089 (9)	−0.0002 (9)

### Geometric parameters (Å, °)

**Table:**

O1—C1	1.360 (3)	C12—H12B	0.9900
O1—C4	1.364 (3)	C13—C14	1.518 (3)
O2—C6	1.202 (3)	C13—C18	1.597 (3)
O3—C6	1.353 (3)	C13—H13	1.0000
O3—C5	1.461 (3)	C14—C15	1.510 (3)
O4—C17	1.217 (3)	C15—C16	1.550 (3)
O5—C22	1.427 (3)	C15—H15A	0.9900
O5—H5	0.84 (1)	C15—H15B	0.9900
O6—C26	1.198 (3)	C16—C17	1.513 (3)
O7—C26	1.330 (3)	C16—C22	1.552 (3)
O7—C27	1.447 (3)	C16—H16	1.0000
O8—C28	1.206 (3)	C17—C18	1.533 (3)
O9—C28	1.351 (3)	C18—C19	1.540 (3)
O9—C25	1.433 (2)	C18—C20	1.576 (3)
C1—C2	1.343 (4)	C19—H19A	0.9800
C1—H1	0.9500	C19—H19B	0.9800
C2—C3	1.438 (3)	C19—H19C	0.9800
C2—H2	0.9500	C20—C25	1.537 (3)
C3—C4	1.350 (3)	C20—C21	1.553 (3)
C3—C5	1.486 (3)	C20—H20	1.0000
C4—H4	0.9500	C21—C24	1.533 (3)
C5—C9	1.548 (3)	C21—C23	1.549 (3)
C5—H5A	1.0000	C21—C22	1.553 (3)
C6—C7	1.497 (3)	C22—H22	1.0000
C7—C8	1.513 (3)	C23—H23A	0.9800
C7—H7A	0.9900	C23—H23B	0.9800
C7—H7B	0.9900	C23—H23C	0.9800
C8—C14	1.333 (3)	C24—H24A	0.9800
C8—C9	1.520 (3)	C24—H24B	0.9800
C9—C11	1.538 (3)	C24—H24C	0.9800
C9—C10	1.539 (3)	C25—C26	1.532 (3)
C10—H10A	0.9800	C25—H25	1.0000
C10—H10B	0.9800	C27—H27A	0.9800
C10—H10C	0.9800	C27—H27B	0.9800
C11—C12	1.521 (3)	C27—H27C	0.9800
C11—H11A	0.9900	C28—C29	1.490 (3)
C11—H11B	0.9900	C29—H29A	0.9800
C12—C13	1.530 (3)	C29—H29B	0.9800
C12—H12A	0.9900	C29—H29C	0.9800
			
C1—O1—C4	105.78 (19)	C17—C16—C22	112.35 (18)
C6—O3—C5	119.61 (16)	C15—C16—C22	115.07 (18)
C22—O5—H5	104 (2)	C17—C16—H16	108.4
C26—O7—C27	114.67 (18)	C15—C16—H16	108.4
C28—O9—C25	117.71 (17)	C22—C16—H16	108.4
C2—C1—O1	111.4 (2)	O4—C17—C16	123.0 (2)
C2—C1—H1	124.3	O4—C17—C18	122.9 (2)
O1—C1—H1	124.3	C16—C17—C18	113.45 (18)
C1—C2—C3	106.0 (2)	C17—C18—C19	108.29 (17)
C1—C2—H2	127.0	C17—C18—C20	109.63 (18)
C3—C2—H2	127.0	C19—C18—C20	115.63 (17)
C4—C3—C2	105.6 (2)	C17—C18—C13	100.00 (16)
C4—C3—C5	126.4 (2)	C19—C18—C13	109.91 (18)
C2—C3—C5	128.0 (2)	C20—C18—C13	112.19 (16)
C3—C4—O1	111.2 (2)	C18—C19—H19A	109.5
C3—C4—H4	124.4	C18—C19—H19B	109.5
O1—C4—H4	124.4	H19A—C19—H19B	109.5
O3—C5—C3	105.74 (16)	C18—C19—H19C	109.5
O3—C5—C9	110.76 (17)	H19A—C19—H19C	109.5
C3—C5—C9	115.06 (18)	H19B—C19—H19C	109.5
O3—C5—H5A	108.4	C25—C20—C21	114.55 (16)
C3—C5—H5A	108.4	C25—C20—C18	113.28 (17)
C9—C5—H5A	108.4	C21—C20—C18	112.67 (16)
O2—C6—O3	118.5 (2)	C25—C20—H20	105.1
O2—C6—C7	123.5 (2)	C21—C20—H20	105.1
O3—C6—C7	117.99 (19)	C18—C20—H20	105.1
C6—C7—C8	117.74 (19)	C24—C21—C23	106.33 (18)
C6—C7—H7A	107.9	C24—C21—C20	111.93 (17)
C8—C7—H7A	107.9	C23—C21—C20	115.78 (18)
C6—C7—H7B	107.9	C24—C21—C22	108.43 (18)
C8—C7—H7B	107.9	C23—C21—C22	107.51 (17)
H7A—C7—H7B	107.2	C20—C21—C22	106.60 (16)
C14—C8—C7	121.15 (19)	O5—C22—C16	108.69 (17)
C14—C8—C9	124.17 (19)	O5—C22—C21	112.06 (17)
C7—C8—C9	114.66 (17)	C16—C22—C21	112.18 (18)
C8—C9—C11	110.70 (16)	O5—C22—H22	107.9
C8—C9—C10	109.21 (18)	C16—C22—H22	107.9
C11—C9—C10	111.35 (18)	C21—C22—H22	107.9
C8—C9—C5	106.23 (16)	C21—C23—H23A	109.5
C11—C9—C5	108.21 (17)	C21—C23—H23B	109.5
C10—C9—C5	111.03 (17)	H23A—C23—H23B	109.5
C9—C10—H10A	109.5	C21—C23—H23C	109.5
C9—C10—H10B	109.5	H23A—C23—H23C	109.5
H10A—C10—H10B	109.5	H23B—C23—H23C	109.5
C9—C10—H10C	109.5	C21—C24—H24A	109.5
H10A—C10—H10C	109.5	C21—C24—H24B	109.5
H10B—C10—H10C	109.5	H24A—C24—H24B	109.5
C12—C11—C9	112.46 (17)	C21—C24—H24C	109.5
C12—C11—H11A	109.1	H24A—C24—H24C	109.5
C9—C11—H11A	109.1	H24B—C24—H24C	109.5
C12—C11—H11B	109.1	O9—C25—C26	111.32 (17)
C9—C11—H11B	109.1	O9—C25—C20	109.29 (16)
H11A—C11—H11B	107.8	C26—C25—C20	112.31 (17)
C11—C12—C13	114.02 (17)	O9—C25—H25	107.9
C11—C12—H12A	108.7	C26—C25—H25	107.9
C13—C12—H12A	108.7	C20—C25—H25	107.9
C11—C12—H12B	108.7	O6—C26—O7	124.9 (2)
C13—C12—H12B	108.7	O6—C26—C25	122.11 (19)
H12A—C12—H12B	107.6	O7—C26—C25	112.95 (18)
C14—C13—C12	112.65 (17)	O7—C27—H27A	109.5
C14—C13—C18	108.77 (17)	O7—C27—H27B	109.5
C12—C13—C18	117.01 (18)	H27A—C27—H27B	109.5
C14—C13—H13	105.9	O7—C27—H27C	109.5
C12—C13—H13	105.9	H27A—C27—H27C	109.5
C18—C13—H13	105.9	H27B—C27—H27C	109.5
C8—C14—C15	122.6 (2)	O8—C28—O9	122.2 (2)
C8—C14—C13	123.70 (19)	O8—C28—C29	126.9 (2)
C15—C14—C13	113.74 (17)	O9—C28—C29	110.79 (19)
C14—C15—C16	113.10 (17)	C28—C29—H29A	109.5
C14—C15—H15A	109.0	C28—C29—H29B	109.5
C16—C15—H15A	109.0	H29A—C29—H29B	109.5
C14—C15—H15B	109.0	C28—C29—H29C	109.5
C16—C15—H15B	109.0	H29A—C29—H29C	109.5
H15A—C15—H15B	107.8	H29B—C29—H29C	109.5
C17—C16—C15	103.94 (19)		
			
C4—O1—C1—C2	0.9 (3)	C15—C16—C17—C18	−68.0 (2)
O1—C1—C2—C3	−0.4 (3)	C22—C16—C17—C18	57.1 (2)
C1—C2—C3—C4	−0.3 (3)	O4—C17—C18—C19	17.1 (3)
C1—C2—C3—C5	177.9 (2)	C16—C17—C18—C19	−171.89 (18)
C2—C3—C4—O1	0.9 (3)	O4—C17—C18—C20	144.1 (2)
C5—C3—C4—O1	−177.3 (2)	C16—C17—C18—C20	−44.9 (2)
C1—O1—C4—C3	−1.1 (3)	O4—C17—C18—C13	−97.9 (2)
C6—O3—C5—C3	−166.70 (18)	C16—C17—C18—C13	73.1 (2)
C6—O3—C5—C9	−41.4 (2)	C14—C13—C18—C17	−62.5 (2)
C4—C3—C5—O3	−140.9 (2)	C12—C13—C18—C17	168.46 (18)
C2—C3—C5—O3	41.3 (3)	C14—C13—C18—C19	−176.26 (17)
C4—C3—C5—C9	96.5 (3)	C12—C13—C18—C19	54.7 (2)
C2—C3—C5—C9	−81.3 (3)	C14—C13—C18—C20	53.6 (2)
C5—O3—C6—O2	177.6 (2)	C12—C13—C18—C20	−75.4 (2)
C5—O3—C6—C7	−5.1 (3)	C17—C18—C20—C25	−147.54 (17)
O2—C6—C7—C8	−155.4 (2)	C19—C18—C20—C25	−24.8 (2)
O3—C6—C7—C8	27.5 (3)	C13—C18—C20—C25	102.3 (2)
C6—C7—C8—C14	−179.1 (2)	C17—C18—C20—C21	−15.5 (2)
C6—C7—C8—C9	−0.7 (3)	C19—C18—C20—C21	107.2 (2)
C14—C8—C9—C11	19.3 (3)	C13—C18—C20—C21	−125.63 (18)
C7—C8—C9—C11	−159.00 (18)	C25—C20—C21—C24	−46.3 (2)
C14—C8—C9—C10	−103.6 (2)	C18—C20—C21—C24	−177.77 (17)
C7—C8—C9—C10	78.1 (2)	C25—C20—C21—C23	75.7 (2)
C14—C8—C9—C5	136.6 (2)	C18—C20—C21—C23	−55.7 (2)
C7—C8—C9—C5	−41.7 (2)	C25—C20—C21—C22	−164.74 (17)
O3—C5—C9—C8	63.6 (2)	C18—C20—C21—C22	63.8 (2)
C3—C5—C9—C8	−176.52 (18)	C17—C16—C22—O5	−129.80 (18)
O3—C5—C9—C11	−177.45 (16)	C15—C16—C22—O5	−11.1 (2)
C3—C5—C9—C11	−57.6 (2)	C17—C16—C22—C21	−5.3 (2)
O3—C5—C9—C10	−55.0 (2)	C15—C16—C22—C21	113.3 (2)
C3—C5—C9—C10	64.9 (2)	C24—C21—C22—O5	−49.9 (2)
C8—C9—C11—C12	−45.1 (2)	C23—C21—C22—O5	−164.50 (18)
C10—C9—C11—C12	76.6 (2)	C20—C21—C22—O5	70.7 (2)
C5—C9—C11—C12	−161.11 (17)	C24—C21—C22—C16	−172.51 (17)
C9—C11—C12—C13	55.3 (2)	C23—C21—C22—C16	72.9 (2)
C11—C12—C13—C14	−35.6 (3)	C20—C21—C22—C16	−51.8 (2)
C11—C12—C13—C18	91.5 (2)	C28—O9—C25—C26	90.9 (2)
C7—C8—C14—C15	−2.3 (3)	C28—O9—C25—C20	−144.50 (18)
C9—C8—C14—C15	179.45 (19)	C21—C20—C25—O9	−47.2 (2)
C7—C8—C14—C13	177.06 (19)	C18—C20—C25—O9	84.0 (2)
C9—C8—C14—C13	−1.2 (3)	C21—C20—C25—C26	76.9 (2)
C12—C13—C14—C8	8.9 (3)	C18—C20—C25—C26	−151.96 (17)
C18—C13—C14—C8	−122.5 (2)	C27—O7—C26—O6	−3.3 (4)
C12—C13—C14—C15	−171.68 (18)	C27—O7—C26—C25	179.2 (2)
C18—C13—C14—C15	56.9 (2)	O9—C25—C26—O6	174.3 (2)
C8—C14—C15—C16	127.9 (2)	C20—C25—C26—O6	51.4 (3)
C13—C14—C15—C16	−51.6 (3)	O9—C25—C26—O7	−8.1 (3)
C14—C15—C16—C17	52.2 (2)	C20—C25—C26—O7	−131.01 (19)
C14—C15—C16—C22	−71.1 (2)	C25—O9—C28—O8	−1.2 (3)
C15—C16—C17—O4	103.1 (2)	C25—O9—C28—C29	177.21 (17)
C22—C16—C17—O4	−131.9 (2)		

### Hydrogen-bond geometry (Å, °)

**Table:**

*D*—H···*A*	*D*—H	H···*A*	*D*···*A*	*D*—H···*A*
O5—H5···O8^i^	0.84 (1)	1.99 (1)	2.827 (2)	175 (3)

Symmetry codes: (i) *x*, *y*+1, *z*.
